# Deficiency of histone variant macroH2A1.1 is associated with sexually dimorphic obesity in mice

**DOI:** 10.1038/s41598-023-46304-8

**Published:** 2023-11-05

**Authors:** Valentina Chiodi, Francesca Rappa, Oriana Lo Re, George N. Chaldakov, Benjamin Lelouvier, Vincenzo Micale, Maria Rosaria Domenici, Manlio Vinciguerra

**Affiliations:** 1 National Centre for Drug Research and Evaluation, Istituto Superiore di Sanita’, Rome, Italy; 2 Department of Biomedicine, Neurosciences and Advanced Diagnostics (BiND), University of Palermo, Palermo, Italy; 3 Department of Translational Stem Cell Biology, Research Institute of the Medical University, Varna, Bulgaria; 4 International Clinical Research Center (FNUSA-ICRC), St’Anne University Hospital, Brno, Czech Republic; 5 Department of Anatomy and Cell Biology, Research Institute of the Medical University, Varna, Bulgaria; 6 Vaiomer, Labège, France; 7 Department of Biomedical and Biotechnological Sciences, Section of Pharmacology, University of Catania, Catania, Italy; 8 Liverpool Centre for Cardiovascular Science (LCCS), Liverpool John Moores University, Liverpool, UK

**Keywords:** Cell biology, Molecular biology, Physiology, Gastroenterology, Molecular medicine

## Abstract

Obesity has a major socio-economic health impact. There are profound sex differences in adipose tissue deposition and obesity-related conditions. The underlying mechanisms driving sexual dimorphism in obesity and its associated metabolic disorders remain unclear. Histone variant macroH2A1.1 is a candidate epigenetic mechanism linking environmental and dietary factors to obesity. Here, we used a mouse model genetically depleted of macroH2A1.1 to investigate its potential epigenetic role in sex dimorphic obesity, metabolic disturbances and gut dysbiosis. Whole body macroH2A1 knockout (KO) mice, generated with the Cre/loxP technology, and their control littermates were fed a high fat diet containing 60% of energy derived from fat. The diet was administered for three months starting from 10 to 12 weeks of age. We evaluated the progression in body weight, the food intake, and the tolerance to glucose by means of a glucose tolerance test. Gut microbiota composition, visceral adipose and liver tissue morphology were assessed. In addition, adipogenic gene expression patterns were evaluated in the visceral adipose tissue. Female KO mice for macroH2A1.1 had a more pronounced weight gain induced by high fat diet compared to their littermates, while the increase in body weight in male mice was similar in the two genotypes. Food intake was generally increased upon KO and decreased by high fat diet in both sexes, with the exception of KO females fed a high fat diet that displayed the same food intake of their littermates. In glucose tolerance tests, glucose levels were significantly elevated upon high fat diet in female KO compared to a standard diet, while this effect was absent in male KO. There were no differences in hepatic histology. Upon a high fat diet, in female adipocyte cross-sectional area was larger in KO compared to littermates: activation of proadipogenic genes (ACACB, AGT, ANGPT2, FASN, RETN, SLC2A4) and downregulation of antiadipogenic genes (AXIN1, E2F1, EGR2, JUN, SIRT1, SIRT2, UCP1, CCND1, CDKN1A, CDKN1B, EGR2) was detected. Gut microbiota profiling showed increase in Firmicutes and a decrease in Bacteroidetes in females, but not males, macroH2A1.1 KO mice. MacroH2A1.1 KO mice display sexual dimorphism in high fat diet-induced obesity and in gut dysbiosis, and may represent a useful model to investigate epigenetic and metabolic differences associated to the development of obesity-associated pathological conditions in males and females.

## Introduction

In the last 20 years, the prevalence of type 2 diabetes (T2D) and of overweight/obesity has dramatically augmented throughout the globe, achieving the status of a pandemic. Epidemiological evidence showed an increased prevalence of T2D in men compared to women^1^. Obese women display an overall greater total body fat content in comparison to men^2^. Importantly, females differ with respect to distribution of adipose tissues, males tend to accrue more visceral fat, whereas females accrue more fat in the subcutaneous depot prior to menopause, a feature associated with protection from the negative consequences associated with obesity and the metabolic syndrome^2,3^. Consistent with humans, young male mice have lower insulin sensitivity than female mice^4^ and are more susceptible to high fat diet (HFD) induced obesity and metabolic syndrome^5,6^. However, the underlying mechanisms that drive sexual dimorphism in metabolism remain unclear.

Several transcriptomic studies in mice showed consistently that hundreds of genes showed sexual dimorphism in liver, adipose, heart, muscle, and in brain in health and upon metabolic/dietary challenge^7–10^. In turn, gene transcriptional activity is controlled by epigenetic mechanisms, ultimately governing the development and the pathophysiology of an organism. The epigenome includes DNA methylation, histone modifications, histone variants, RNA-mediated processes, and disruption of epigenomic balance may cause several pathologies including obesity and T2D. In this context, DNA methylation is the most extensively studied epigenetic mechanism^11^. Epigenetic mechanisms, by linking environmental factors to altered gene activity, represents an obvious link between the rapid change in eating habits and the observed obesity phenotypes. For instance, obesity is associated with differential DNA methylation and increased epigenetic variability^11,12^. Moreover, different diets and nutrients are associated with differential DNA methylation in human tissues including adipose tissue, skeletal muscle, and pancreatic islets^11^. It has also been reported that DNA methylation differs between sexes, and this has been linked to the development of cardiometabolic diseases and to the impact of maternal obesity and of gestational diabetes in the offspring^13,14^. The evidence regarding DNA methylation and sexual dimorphism of obesity and T2D has a correlative nature. Moreover, the contribution of other epigenetic mechanisms, notably the role of histone variants, in the sexual dimorphism of obesity and T2D has not been explored.

In addition to canonical histones (H1, H2A, H2B, H3 and H4), histone variants replace replication-coupled histones in a subset of nucleosomes, conferring chromatin unique properties to modulate gene expression^15^. Histone variants have specific genomic distribution, they are regulated by ad hoc deposition and removal machineries and have important roles in development, nutrient sensing and cell plasticity^15^. MacroH2A1 is an evolutionary conserved variant of histone H2A, coded by the gene *H2AF*Y, that regulates nutrient metabolism, cell plasticity, proliferation and senescence^11,12^. MacroH2A1 is present in two alternatively exon-spliced isoforms, macroH2A1.1 and macroH2A1.2, which have both opposite and redundant roles^16,17^. MacroH2A1 was originally described as enriched on the inactive X chromosome (Xi) in mammals and on the facultative heterochromatin of autosomes, and detected in the senescence-associated heterochromatin foci; hence it was long believed to be an exclusive repressive marker. However, it has been shown also to play a positive role in the expression of genes according to the context and to the gene body region of binding^17,18^. The structural and functional requirements for deposition of macroH2A1 into distinct chromatin domains recently began to be elucidated^19–23^. In in vitro models of non alcoholic fatty liver disease (NAFLD), the hepatic manifestation of obesity, overexpression of macroH2A1.1 led to a reduced lipid accumulation, while macroH2A1.2 overexpression yielded opposite effects^24–26^. In a limited case series of morbidly obese patients, the expression of macroH2A1.1 was specifically and massively increased in the adipose tissue, correlating with the BMI^27^. The histone variant macroH2A1.1 contains a macrodomain capable of binding NAD^+^-derived metabolites, which is not present in macroH2A1.2, which is thought to mediate some of its specific cellular functions such as gene expression and modulation of mitochondrial respiration^28,29^. We recently generated a mouse model where the histone variant isoform macroH2A1.1, but not macroH2A1.2, was depleted in the whole body^30^. These mice appear normal under unchallenged circumstances. However, they present a phenotype at the level of social behavior^30,31^, without remarkable sex specific differences. A few previous independent studies using mice genetically depleted for the whole macroH2A1 gene (without making a distinction between the isoforms macroH2A1.1 and macroH2A1.2) have shown changes in glucose tolerance^32,33^ in males, and exacerbated hepatic steatosis in females^34^. The role of single macroH2A1 isoforms in obesity-related sexual dimorphism has never been investigated. In this work, by using the above mentioned mouse model genetically depleted of macroH2A1.1^30^, we investigated its potential epigenetic role in sexual dimorphic obesity and its associated metabolic disturbances such as gut dysbiosis, by feeding males and females with a long-term obesogenic high fat dietary regimen.

## Materials and methods

### Mice

Mice lacking macroH2A1.1 were generated as follows^30^: a 12 kb segment of the murine H2AFY gene (introns 5–8) was subcloned from a BAC by recombineering into p15A-HSV tk-DTA-amp. A lacZ-neo cassette^35^, flanked by loxP and rox sites at the 5′and 3′ ends, respectively, was inserted into the intron between exons 6a and 6b, also by recombineering. Another rox site was inserted upstream of exon 6a and another loxP site inserted downstream of exon 6b so that Dre/rox recombination would remove exon 6a and the lacZ-neo cassette, and Cre/loxP recombination would remove exon 6b and the lacZ-neo cassette^35^; thus, Cre recombination will eliminate macroH2A1.1 expression. Southern blotting of genomic NheI-digested DNA from individual ES-cell-derived clones with a 3’ probe was used to identify homologous recombinants^30^. A 12.3-kb DNA fragment corresponds to the wild-type macroH2A1.1 locus; integration of the loxP-flanked neomycin cassette 3’ of exon 6b introduced an additional NheI site, thus increasing the size of the NheI DNA fragment to 16.2 kb in the targeted allele^30^. Cre-mediated recombination resulted in a 3.9-kb NheI DNA fragment recognized by the 3′ probe, which is diagnostic of the macroH2A1.1 allele. The targeting of the macroH2A1.1 allele was performed by electroporation of A9 ES cells, which were then injected into C57BL/6 eight cell-stage embryos. The targeted macroH2A1.1^Fl/Fl^ mice were crossed to deleter HPRT-Cre mice (129S1/Sv-Hprttm1(CAG-cre)Mnn/J), purchased from Jackson Laboratories, USA, to remove the loxP-flanked neomycin cassette and generate macroH2A1.1^Fl/−^ mice (heterozygous, HET). Mice heterozygous for the macroH2A1.1 allele were further crossed to deleter Cre mice to generate the macroH2A1.1^−/−^ (knockout, KO) mice. All mice used were obtained after eight generations of back crossing on a C57BL/6 genetic background. Mice were bred and maintained at Plaisant Srl (Rome, Italy). In accordance with current European and Italian legislation (2010/63/EU, DI 26/2014), all animal procedures were approved by the Ethical Committee (OPBA) of the Italian National Health Institute (Rome, Italy) and by the Italian Ministry of Health (specific authorization n° 730/2020- PR). The authors complied with the ARRIVE guidelines.

### Diet and food intake

Mice of the two genotypes (macroH2A1.1^Fl/Fl^ and macroH2A1.1^−/−^: Fl/Fl and KO, respectively) were housed 2 animals per cages in environmentally controlled conditions: 22 ± 1 °C temperature and 50% humidity, with 12 h light/12 h dark, (dark phase started at 7.00 pm). After 2 weeks of acclimatization, male and female Fl/Fl and KO mice were randomly assigned to two groups: animals fed with a standard diet (SD) and animals fed with a high fat diet (HFD). Male and females animals were analyzed separately.

The diet consisted of a purified high fat diet used to induce obesity, containing 60% of energy derived from fat (Teklad, TD 06414, Envigo; 36% saturated, 41% monounsaturated, 23% polyunsaturated; 18% protein). This diet allows mice to gain weight consistently and it is frequently used to induce obesity^36–38^.

The diet was administered for three months starting from 10 to 12 weeks of age. Food consumption and body weight were measured twice a week, starting one week before the diet, until the end of the treatment. Food was changed biweekly; food intake was assessed by subtracting remaining food, including food spilled in the cage, from a weighed aliquot. Glucose Tolerance Test (GTT) was performed as previously described^39,40^. The area under the curve (AUC) was used to quantify the total increase in blood glucose during the GTT. For scarification / blood sampling, isoflurane anesthesia was used.

### Histological analyses

Paraffin-embedded sections of the liver and white adipose tissue (4 μm) were processed by haematoxylin and eosin for histological evaluation, as described previously^41,42^. Diagnostic classification of NAFLD was performed by applying a semiquantitative scoring system that grouped histological features into broad categories (steatosis, hepatocellular injury, portal inflammation, fibrosis and miscellaneous features)^43^. The white adipose tissue sections were observed with an optical microscope (Microscope Axioscope 5/7 KMAT, Carl Zeiss, Milan, Italy) connected to a digital camera (Microscopy Camera Axiocam 208 color, Carl Zeiss, Milan, Italy) for evaluation.

### Gene expression

Total RNA was isolated from cells using TRIzol (Invitrogen), and from formalin‐fixed paraffin embedded samples of mice white adipose tissue using the RNeasy FFPE kit (Qiagen, Italy). RNA was quantified using a NanoDrop 1000 spectrophotometer (Thermo Scientific). Samples with an RNA integrity number between 9 and 10 were used for cDNA preparation (100 ng to 1 μg per sample). A commercially available adipogenesis array [mouse RT^2^ Profiler PCR Array (96-Well Format) for Mouse Adipogenesis, Cat. no. 330231 PAMM-049ZA, Qiagen, Italy] was used to measure genes involved in adipogenesis by qRT‐PCR in mice white adipose tissue; expression data were normalized to the geometric mean of three house keeping genes (Actb, GAPDH and GusB). Genes were considered significantly differentially expressed if their False Discovery Rate (FDR)-adjusted P value^44^ was < 0.01 and their absolute log2 fold-change was ≥ 1 (fold-change 2).

### Metagenomic profiling

The microbial population present in the fecal samples from mice was determined using next generation high throughput sequencing of variable regions of the 16S rRNA bacterial gene. The workflow performed at VAIOMER (France) includes the steps of (i) DNA extraction, (ii) Library construction and sequencing, (iii) bioinformatics and figure generation.

Briefly, after extraction of the feces sample using QIAamp Fast DNA Stool Mini Kit (Qiagen, Hilden, Germany), PCR amplification was performed using 16S universal primers targeting the V3‐V4 region of the bacterial 16S ribosomal gene (Vaiomer universal 16S primers). The joint pair length was set to encompass 476 base pairs amplicon thanks to 2 × 300 paired‐end MiSeq kit V3 (Illumina, San Diego, CA, USA). For each sample, a sequencing library was generated by addition of sequencing adapters. The detection of the sequencing fragments was performed using MiSeq Illumina® technology. The targeted metagenomic sequences from microbiota were analyzed using a proprietary bioinformatics pipeline established by Vaiomer (FROGS v1.4.0). In brief, after demultiplexing of barcoded Illumina paired reads, single-read sequences were cleaned and paired into longer fragments. OTUs were produced with single-linkage clustering (swarm algorithm v2.1.6). The average number of raw read pairs was ~ 41,000. The average number of read pairs classified in OTU is ~ 22,500. The proportion of discarded sequences between raw and classified read pairs is ~ 45%, which is a standard and acceptable value; the reads counts for each sample is shown in Supplemental Fig. 1. The taxonomic assignment was performed by BLAST (Blast+v2.2.30+) against SILVA 132 Parc database to determine bacterial profiles from phylum to genus, and when reachable to species level. The following specific filters were applied for this analysis to obtain the best results: (1) the last 10 bases of reads R1 were removed; (2) the last 50 bases of reads R2 were removed; (3) the following filters were applied: amplicons with a length of < 350 nt or a length of > 490 nt were removed and (4) OTUs with abundance lower than 0.005% and that appear less than twice in the entire dataset were removed. Alpha and beta diversity analyses were conducted on the OTU table (Scikit-Bio v0.4.2).LEfSe method: The Operational Taxonomic Unit (OTU) files generated were uploaded and formatted for LEfSe analysis using the per‐sample normalization of sum values option. The linear discriminant analysis effect size was determined using default values (alpha value of 0.5 for both the factorial Kruskal‐Wallis test among classes and the pairwise Wilcoxon test between subclasses, threshold of 2.0 for the logarithmic LDA score for discriminative features) and the strategy for multi‐class analysis set to ‘all against‐all’. LEfSe cladograms from the LDS effect size data were generated with Bacteria as the tree root.

**Figure 1:**
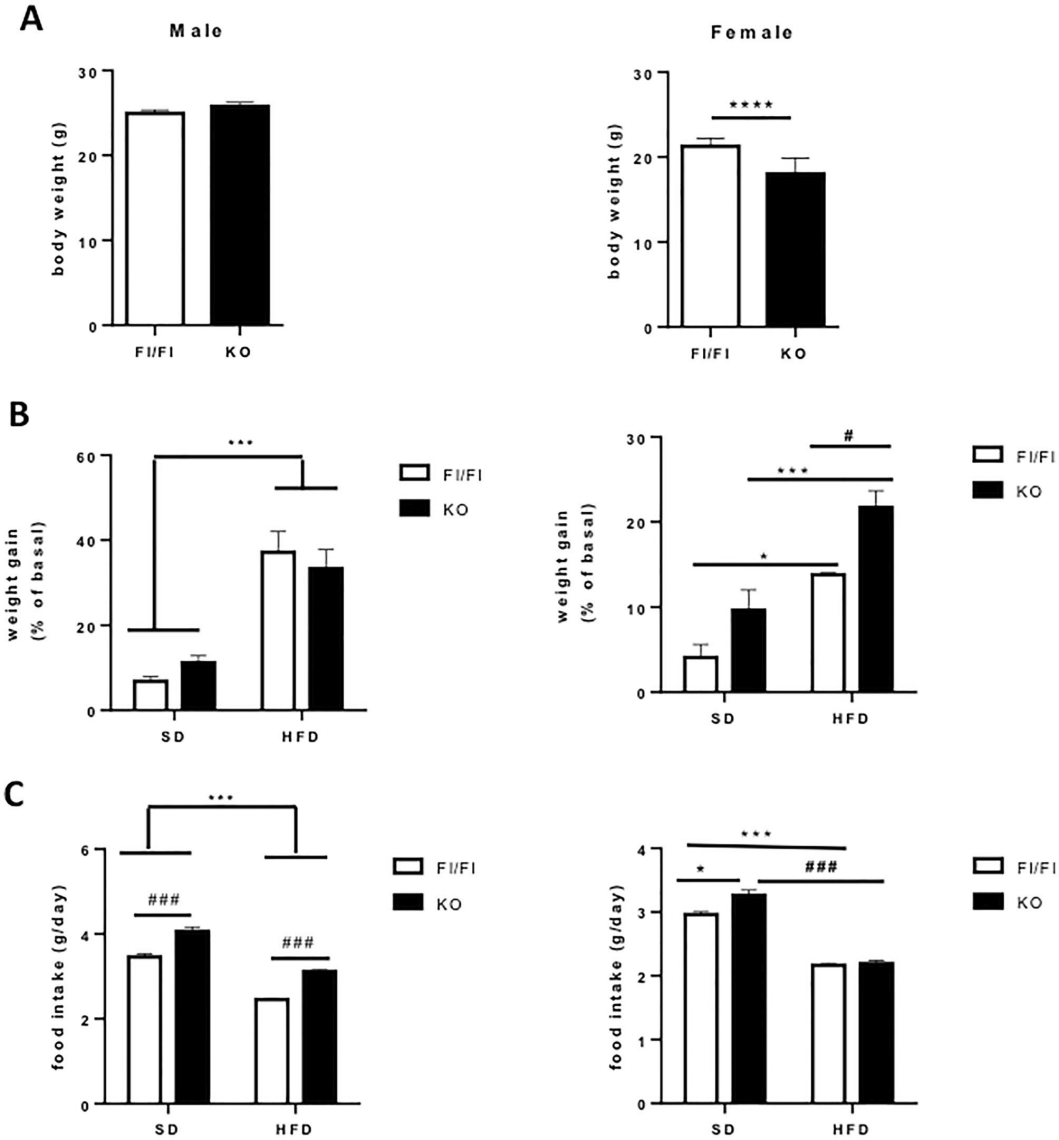
Effects of high fat diet (HFD) in Fl/Fl and macroH2A1.1 KO mice. (**A**) Basal body weight (in g) of mice at the beginning of the experiments. (**B**) Body weight gain (percentage increase with respect to basal body weight) of mice under standard diet (SD) or HFD in the Fl/Fl or KO background, divided by sex. (**C**) Food intake per day (g) in mice under standard diet (SD) or HFD in the Fl/Fl or KO background, divided by sex. Data are presented as means ± S.E.M (n = 6–12 mice/group). ^*,#^*p* < 0.05, ***^,###^*P* < 0.001, ****p < 0.0001, comparison as indicated, determined by unpaired t-test or two-way ANOVA test.

### Statistical analyses

The results are presented as group mean ± SEM. Data were analysed using one- or two-way ANOVA (factor 1: diet; factor 2: genotype) followed by post-hoc Fisher’s LSD for multiple comparisons, if appropriate. Unpaired t-test was used to analyze independent data (Fl/Fl vs. KO). Statistical evaluations were performed using specialized software (Graph-Pad Prism 9.0, San Diego, CA, USA). A *p* level of 0.05 or less was considered indicative of a significant difference.

### Ethical approval

All animal procedures were approved by the Ethical Committee (OPBA) of the Italian National Health Institute (Rome, Italy) and by the Italian Ministry of Health (specific authorization n° 730/2020- PR).

## Results

### Genetic depletion of macroH2A1.1 aggravates high fat diet (HFD)-induced obesity and glucose intolerance in female mice

Previous studies using systemically KO mice for the whole macroH2A1 gene have shown mild glucose intolerance in males^32^ and hepatic lipid accumulation in females^34^ in non obesogenic conditions. To investigate the potential epigenetic role specifically of macroH2A1.1 isoform in sex dimorphic obesity and metabolic disturbances, we challenged wild type (Fl/Fl) or macroH2A1.1 knockout (KO) male and female animals with a long-term high fat dietary regimen. We adopted a well-established mouse model of obesity, where young mice (10–12 weeks of age) are fed a HFD (60% energy from lard)^36–38^ for 12 weeks, after which the animals were sacrificed for further analyses. Interestingly, in male mice there was not difference in basal body weight between Fl/Fl and KO mice (t = 1.240, *p* = 0.3095) (Fig. 1A, left panel). Instead, female KO mice had lower body weight than Fl/Fl mice (t = 5.650, *p* < 0.0001) (Fig. 1A, right panel). Eight groups of 6–12 mice each were then created: male Fl/Fl mice fed a SD; male Fl/Fl mice fed a HFD; female Fl/Fl mice fed a SD, female Fl/Fl mice fed a HFD; male KO mice fed a SD; male KO mice fed a HFD; female KO mice fed a SD; female KO mice fed a HFD. At the end of the dietary regimen, two-way ANOVA revealed for male mice a significant effect of diet on body weight gain (F1, 33 = 51.12, *p* < 0.0001), but neither a main genotype effect (F1, 33 = 0.0054, *p* = 0.9415) nor a diet x genotype interaction (F1, 33 = 1.269, *p* = 0.2681), suggesting that HFD induced a significantly higher increase in body weight both in Fl/Fl and KO mice, without differences between the genotypes (Fig. 1B, Supplementary Table 1).

In female mice, two-way ANOVA revealed a significant effect of diet (F1, 29 = 22.60, *p* < 0.0001), a main genotype effect (F1, 29 = 8.657, *p* = 0.0063), but not a diet x genotype interaction (F1, 29 = 0.2645, *p* = 0.6109) (Fig. 1B, Supplementary Table 1). Post-hoc analysis revealed that HFD significantly increased the body weight of Fl/Fl (*p* < 0.05 vs. Fl/Fl SD) and KO mice (*p* < 0.001 vs. KO SD) but the weight gain induced by HFD was significantly more pronounced in KO than in Fl/Fl (*p* < 0.05) (Fig. 1B, Supplementary Table 1). These results suggest that macroH2A1.1 deletion may affect weight gain related to dietary regimen based on sex difference.

Regarding food intake, in female mice two-way ANOVA revealed a significant effect of diet (F1, 143 = 198.9, *p* < 0.0001), a main genotype effect (F1, 143 = 6.144, *p* = 0.00143), and a diet x genotype interaction (F1, 143 = 4.357, *p* = 0.0386) (Fig. 1C, Supplementary Table 1). Post-hoc analysis revealed that the food intake was significantly less in mice fed with HFD as compared to SD, irrespective of genotype (*p* < 0.0001, Fig. 1C, Supplementary Table 1). Furthermore, when fed with SD KO mice showed higher food intake than the Fl/Fl (*p* < 0.05), while no difference between the two groups was described when animals were fed with HFD (*p* > 0.05) (Fig. 1C, Supplementary Table 1). In male mice, two-way ANOVA revealed a significant effect of diet (F1, 218 = 258.2, *p* < 0.0001), a main genotype effect (F1, 218 = 108.9, *p* < 0.0001), but not a diet x genotype interaction (F1, 218 = 0.2362, *p* = 0.6275), suggesting that HFD significantly reduced food intake both in Fl/Fl and in KO mice and that food intake was higher in KO mice, independently of the diet.

**Figure 2:**
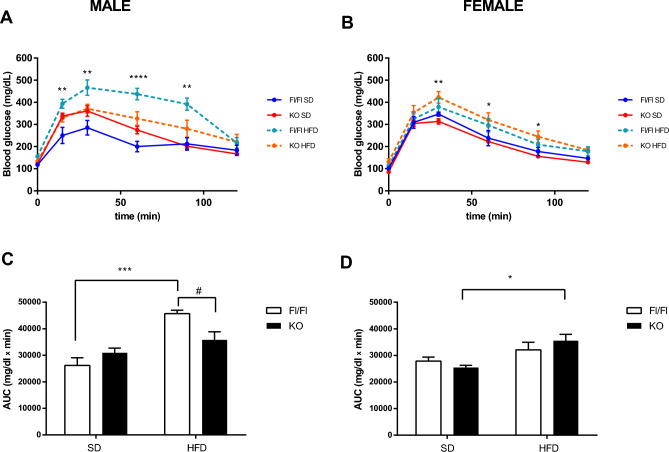
Responsiveness to glucose in Fl/Fl and macroH2A1.1 KO mice, according to the sex. Glucose tolerance test (GTT) was performed in Fl/Fl and macroH2A1.1 KO mice fed a standard diet (SD) or a high fat diet (HFD) following a 6 h fast. Mice were treated with glucose 2 g/kg, i.p., and blood glucose concentrations were measured at different time points (**A**–**B**) and AUC (**C**–**D**). Data are expressed as mean ± S.E.M. (n = 6–12 mice/group). *p < 0.05, **p < 0.01, ***p < 0.001, ****p < 0.0001 and #*p* < 0.05, unpaired t-test or two-way ANOVA test.

We then investigated the ability of male and female mice to respond to a glucose challenge. In glucose tolerance tests (GTT), glucose levels were slightly but not significantly elevated upon HFD in females Fl/Fl compared to females Fl/Fl fed a SD, while they were significantly elevated (at 30, 60 and 90 min) upon HFD in females KO (*p* < 0.05 or *p* < 0.01 vs. female KO fed a SD). Similarly, the AUC was significantly increased in KO females HFD (p < 0.01 vs. KO SD) (Two-way ANOVA, factor genotype F1, 27 = 0.05, *p* = 0.82; factor diet F1, 27 = 9.98, *p* = 0.0039 and factor genotype x diet interaction F1, 27 = 1.67, *p* = 0.21) (Fig. 2B–D). Conversely, in Fl/Fl males HFD induced a massive increase in blood glucose levels (at 15, 30, 60 and 90 min) (*p* < 0.05, *p* < 0.01 or *p* < 0.001 vs. males Fl/Fl fed a SD). Similarly, the AUC was significantly increased in Fl/Fl males HFD (*p* < 0.0001 vs. Fl/Fl males SD), but not in KO HFD males as compared to the respective SD group (Two-way ANOVA, factor genotype F1, 27 = 0.96, *p* = 0.33; factor diet F1, 27 = 22.37, *p* < 0.0001 and factor genotype x diet interaction F1, 27 = 8.05, *p* = 0.0085). Interestingly, in males, KO genotype conferred protection against HFD-induced increase in glucose levels (Fig. 2A–C). Altogether these findings suggest that genetic depletion of histone variant macroH2A1.1 aggravates HFD-induced obesity and glucose intolerance in female, but not male, mice.

### Genetic depletion of macroH2A1.1 worsens HFD-induced increase in adipocyte area in female mice

**Figure 3:**
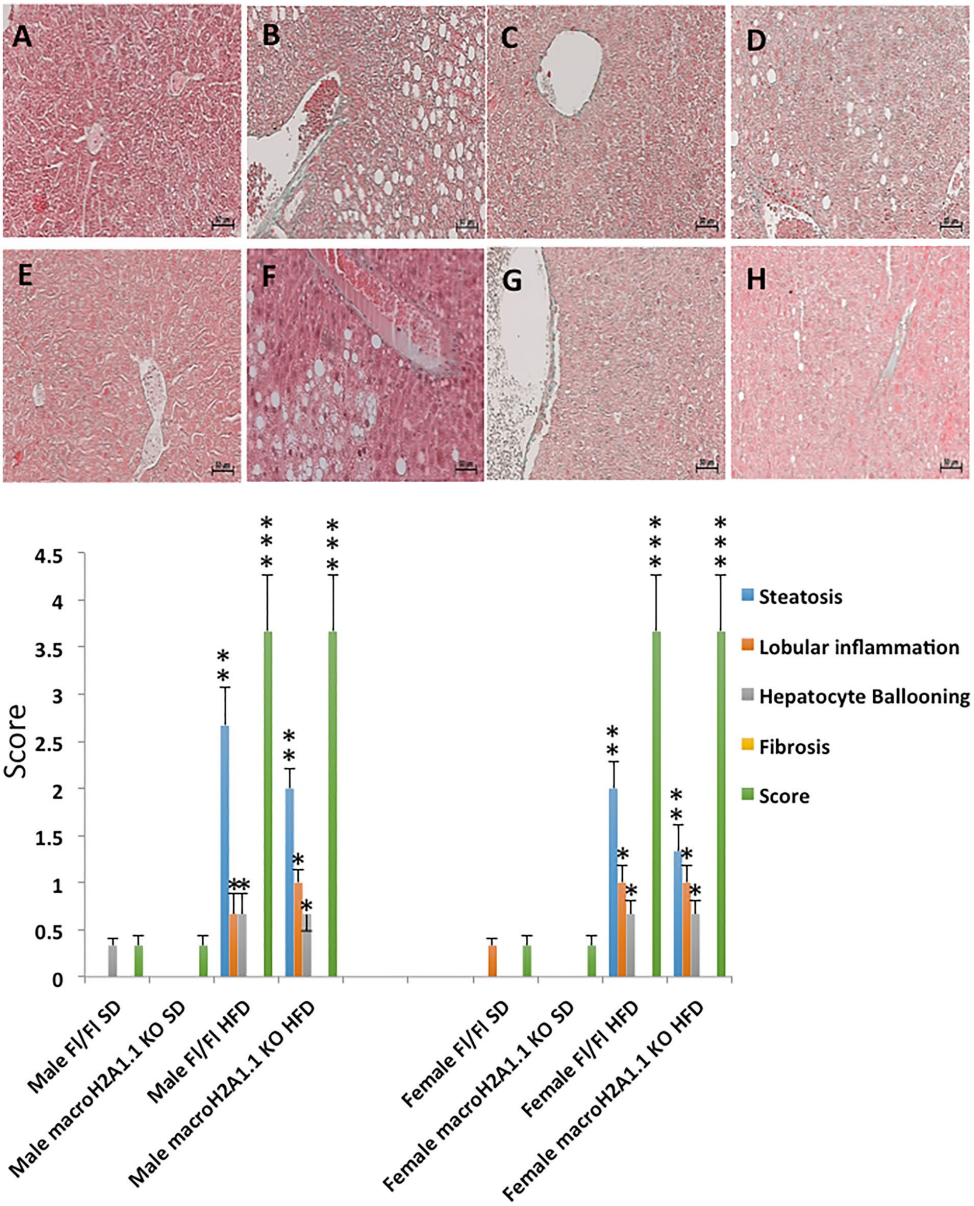
Liver histological analysis. *Upper panels*: representative pictures from hematoxylin and eosin (H&E) staining of liver sections around the lobular areas in Fl/Fl and macroH2A1.1 KO mice fed with standard diet (SD) or with high fat diet (HFD). (**A**) Males Fl/Fl SD; (**B**) Males Fl/Fl HFD; (**C**) Females Fl/Fl SD; (**D**) Females Fl/Fl HFD; (**E**) Males macroH2A1.1 KO SD; (**F**) Males macroH2A1.1 KO HFD; (**G**) Females macroH2A1.1 KO SD; (H) Females macroH2A1.1 KO HFD. Magnification 200x, scale bar 50 µm. *Lower panel*: NAFLD and inflammation were scored using a semiquantitative system that grouped histological features into broad categories (steatosis, hepatocellular injury, portal inflammation, fibrosis and miscellaneous features) in males (left panel) and in females (right panel). Data are expressed as mean ± S.E.M (n = 6–8 mice/group). One-way ANOVA followed by Tukey post-hoc test, *p < 0.05, **p < 0.01, ***p < 0.001, HFD condition versus respective SD condition (i.e. same sex or same genotype).

Obesity is often associated with NAFLD, a condition characterized by parenchymal triglyceride accumulation and inflammation. We sought to analyze the lipid content in the liver of male and female KO versus Fl/Fl mice: H&E and trichromic staining revealed that HFD increased consistently the score for steatosis, lobular inflammation, hepatocyte ballooning and fibrosis compared to a SD, with no evident difference between sexes or between genotypes (Fig. 3).

**Figure 4:**
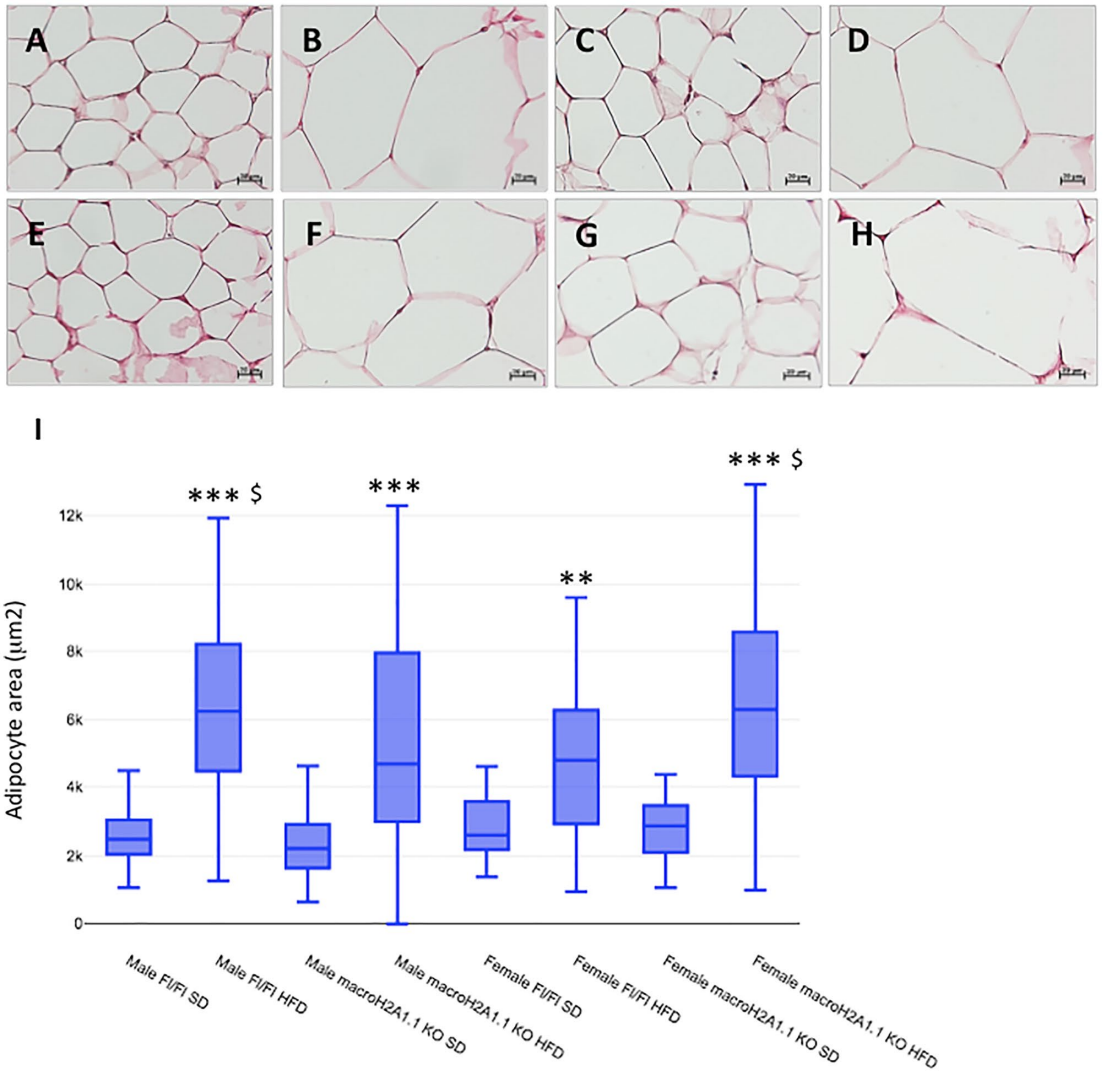
White adipose tissue histological analysis. *Upper panels*: representative pictures from hematoxylin and eosin (H&E) staining of white adipose tissue sections of Fl/Fl and macroH2A1.1 KO mice fed with standard diet (SD) or with high fat diet (HFD). (**A**) Males Fl/Fl SD; (**B**) Males Fl/Fl HFD; (**C**) Females Fl/Fl SD; (**D**) Females Fl/Fl HFD; (**E**) Males macroH2A1.1 KO SD; (**F**) Males macroH2A1.1 KO HFD; (**G**) Females macroH2A1.1 KO SD; (**H**) Females macroH2A1.1 KO HFD. Magnification 400x, scale bar 20 µm. (**I**) Histograms showed the adipocytes area evaluation obtained by Zeiss Software. The adipocytes area is expressed in µm^2^. Data are expressed as mean ± S.E.M. (n = 6–8 per group). One-way ANOVA followed by Tukey post-hoc test, ***p < 0.001, **p < 0.01, HFD condition versus respective SD condition (i.e., same sex, same genotype); ^$^*p* < 0.05 versus female Fl/Fl HFD.

Histological analysis (H&E) of the area of adipocytes within white adipocyte tissue of male Fl/Fl (Fig. 4B vs. 4A) and female Fl/Fl (Fig. 4D vs. 4C) mice, and of male KO (Fig. 4F vs. 4E) and female KO (Fig. 4H vs. 4G), revealed a significant increase upon HFD. Interestingly, macroH2A1.1 KO genotype exacerbated HFD-induced increase adipocyte area in female (Fig. 4H vs. 4F) but not in male Fl/Fl mice (Fig. 4D vs. 4B). Quantifications of adipocyte areas are shown in Fig. 4I.

Obesity is associated mainly with adipose cell hypertrophic enlargement within the visceral fat, the largest fat depot, and this unhealthy expansion promotes the obesity-associated metabolic complications^45^. To study macroH2A1.1-dependent transcriptional effects in the adipose tissue of HFD mice, we used a commercial gene array profiling the expression of 84 key genes involved in white adipose tissue adipogenesis, including hormones, adipokines, enzymes and transcription factors. Using a twofold cutoff difference in mRNA expression upon HFD diet in either of the two groups of animals, we identified 17 genes oppositely regulated in the visceral adipose tissue of female KO compared to Fl/Fl mice (Table 1). Conversely, in males only 7 genes were oppositely regulated in the adipose tissue of KO compared to Fl/Fl mice (Table 1). Pro-differentiation and pro-adipogenic genes ACACB, AGT, ANGPT2, FASN, RETN and SLC2A4 were significantly upregulated in female KO mice fed a HFD while only ANGPT2, FASN, RETN and SLC2A4 were upregulated, to a lesser extent, in male macroH2A1.1 KO mice fed a HFD (Table 1). Conversely, anti-adipogenic genes AXIN1, E2F1, EGR2, JUN, anti-inflammatory genes SIRT1, SIRT2, thermogenic gene UCP1 and proliferation-regulating genes CCND1, CDKN1A, CDKN1B, EGR2 were downregulated in female macroH2A1.1 KO mice fed a HFD (Table 1). In male KO mice, only AXIN1, CDKN1A and EGR2 were similarly downregulated (Table 1). The full list of genes detected is shown in Supplemental Table 2.

**Table 1:** Adipogenic gene expression in male/female macroH2A1.1 KO mice fed a high fat diet (HFD), as compared to their respective Fl/Fl HFD group.

Refseq	Symbol	Female macroH2A1.1 KO + HFD	*P* value	Male macroH2A1.1 KO + HFD	*P* value
		Fold regulation		Fold regulation	
NM_133904	Acacb	**3.054**	0.00012	0.942	0.08712
NM_007428	Agt	**3.405**	0.00022	1.675	0.35953
NM_007426	Angpt2	**14.506**	1.50E−12	**2.007**	0.00434
NM_009733	Axin1	*− 10.932*	6.70E−09	*− 2.056*	0.00703
NM_007631	Ccnd1	*− 3.207*	0.00341	1.678	0.43392
NM_009870	Cdk4	*− 2.807*	0.00416	1.473	0.26307
NM_007669	Cdkn1a	*− 2.792*	0.00945	*− 2.452*	0.00762
NM_009875	Cdkn1b	*− 2.518*	0.00017	1.429	0.55048
NM_007891	E2f1	*− 4.723*	0.00002	1.123	0.06932
NM_010118	Egr2	*− 11.408*	2.45E−08	*− 2.778*	0.00946
NM_007988	Fasn	**8.790**	1.27E−07	**3.530**	0.00033
NM_010591	Jun	*− 2.503*	0.00193	1.502	0.67022
NM_022984	Retn	**4.048**	8.50E−09	**2.654**	0.00028
NM_019812	Sirt1	*− 2.304*	0.00250	1.502	0.35415
NM_022432	Sirt2	*− 2.004*	0.00679	1.399	0.52883
NM_009204	Slc2a4	**4.532**	0.00034	**2.302**	0.00512
NM_009463	Ucp1	*− 5.107*	7.80E−07	1.354	0.43910

Values represent means of fold regulation of 3 mice per condition.

In bold = genes upregulated > 2-fold compared to Fl/Fl mice of the respective gender.

In italic = genes downregulated > 2-fold compared to Fl/Fl mice of the respective gender.

*P* values adjusted for multiple testing using false discovery rate method (< 0.01).

### Gut microbiota profiling by metagenomic sequencing shows enhanced obesity-related dysbiosis in female macroH2A1.1 KO mice

**Figure 5:**
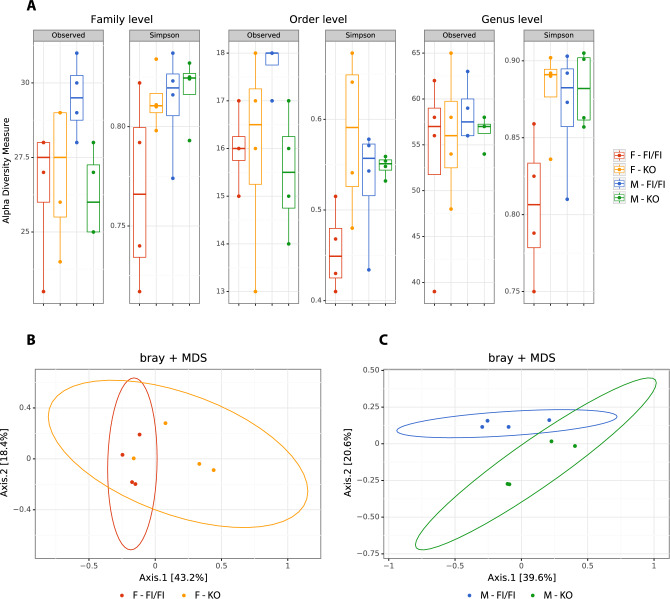
Taxonomic diversity between the gut microbiota of macroH2A1.1 KO versus control (Fl/Fl, males and females). (**A**) The panel represents the alpha diversity at family, order and genus level in the different groups. Observed and Simpson indexes calculate the alpha diversity in terms of richness (number of taxa that are present in the sample). (**B**) Multidimensional scaling (MDS) Ordination on Bray–Curtis Index. (**C**) Hierarchical Clustering on Bray–Curtis Index. (**B**) and (**C**) are two graphical representations of the beta diversity, to visualize the global level of similarity between the sample bacterial profiles based on the Bray–Curtis method.

Established evidence has implicated HFD-induced alterations in gut microbiota in the obesity epidemic. HFD consumption generally leads to a decrease in Bacteroidetes and an increase in Firmicutes, alterations that have been associated with obesity and subsequent development of chronic diseases^46,47^. As macroH2A1.1 KO female mice displayed decreased glucose tolerance, increased body weight and adipocyte area, we sought to identify bacterial populations contained in fecal samples from male and female KO mice using next generation high throughput sequencing of variable regions (V3–V4) of the 16S rDNA bacterial gene^48^. Alpha diversity analyses, representing the mean of species diversity in each sample, showed significant taxonomic differences between female KO and Fl/Fl mice at the order and at the genus level according to Simpson index (Kruskal–Wallis or Wilcoxon Rank Sum test, *p* = 0.0433), and between male KO and Fl/Fl mice at the order and at the family level according to the observed index (Kruskal–Wallis test *p* = 0.028; Wilcoxon Rank Sum test, *p* = 0.03), but not between the two genders (Fig. 5A). Interestingly, alpha diversity was increased in females and decreased in male upon macroH2A1.1 KO. Feces microbial composition was highly different between KO and control mice, as shown by beta diversity metrics based multi-dimensional scaling ordination (Fig. 5B) and by hierarchical clustering (Fig. 5C).

**Figure 6:**
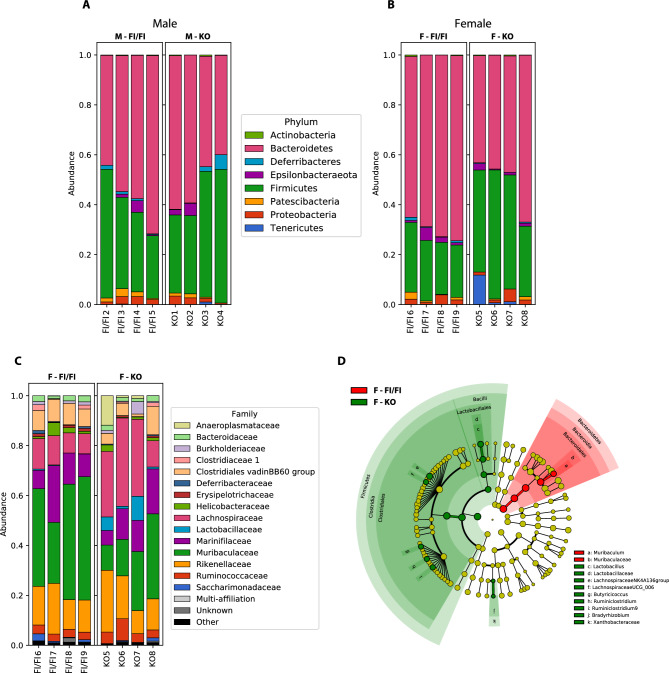
Taxonomic diversity between the gut microbiota of macroH2A1.1 KO versus control (Fl/Fl, males and females). The figures show the taxa relative abundance per sample at Phylum level in male (**A**) and female (**B**) mice, for the two genotypes control (Fl/Fl) and macroH2A1.1 KO. The top 15 bacterial Phyla are visualized. (**C**) Taxa relative abundance per sample at the Family level in female mice, for the two genotypes control (Fl/Fl) and macroH2A1.1 KO. The top 15 bacterial phyla are visualized. (**D**) LefSe result cladogram (genus and above levels) indicates the bacterial taxa that are significantly different between control (Fl/Fl) and macroH2A1.1 KO female mice (*p* value < 0.05, Wilcoxon–Mann–Whitney test).

The community structures observed in the different groups were significantly different. At the phylum level, Firmicutes and Bacteroidetes dominated the fecal microbiota in all groups (Fig. 6A). No differences of Firmicutes and Bacteroidetes relative abundance were observed between KO and control male mice (Fig. 6A). However, an increase in Firmicutes and a decrease in Bacteroidetes were observed in KO compared to Fl/Fl female mice (Fig. 6B). We thus focused our subsequent in depth analyses on female mice. At the family level, the fecal microbiota was dominated by Lachnospiraceae and Muribaculaceae, which were increased and decreased in KO compared to Fl/Fl mice, respectively (Fig. 6C). Focusing on genotype effect in female mice, broad population changes were seen from phylum to family level, and significantly enriched taxa were identified using LDA Effect Size (LEfSe) analysis. Clostridiales and Lactobacillales orders were significantly enriched in macroH2A1.1 KO female mice, while Bacteroidales were mostly overrepresented in control female mice (Figs. 6D), the most striking result of our metagenomic analyses in gut microbiota composition.

## Discussion

The epigenetic mechanisms that drive sexual dimorphism in the development of obesity and of metabolic disturbances are unclear. Variations in the expression of histone variants have been observed in tissues accumulating fat, in mouse models and in human being^49,50^; however, of the ~ 20 histone variants existing in mammals^15^ so far only manipulation of macroH2A1, H2A.Z, H3.3 and H2A.v has been shown to modulate lipid homeostasis and adipogenesis^25–27,32–34,49,51,52^. Here, we have shown for the first time that genetic ablation of large histone variant isoform macroH2A1.1 exacerbated obesity and dysmetabolism induced by a HFD specifically in female, but not in male, mice. Compared to their control genotype, macroH2A1.1 KO female mice fed a HFD developed accrued obesity and glucose intolerance, which was accompanied by adipocyte hypertrophy with pro-adipogenic gene expression. The HFD used in our study contains 60% fat from lard, and it is well established to be obesogenic in adult mice in the C57BL/6 background^36–38^; with an approximate fatty acid profile (% of total fat) of 37% saturated, 47% monounsaturated, 16% polyunsaturated. Obesity-related macroH2A1.1 dependent phenotypes using other common HFD remain to be studied. Expression analysis of selected mRNA transcripts implicated in adipogenesis uncovered upregulation of pro-adipogenic genes ACACB, AGT, FASN, RETN and SLC2A4 and downregulation of anti-adipogenic genes E2F1, EGR2, JUN, anti-inflammatory genes SIRT1, SIRT2, thermogenic gene UCP1 and pro-proliferative genes CCND1, CDKN1A, CDKN1B in female KO mice, which is consistent with an increase of adipocyte mass. However, the use of a focused gene expression panel could introduce bias, compared to a whole transcriptome analysis, which is a more comprehensive and discovery-driven approach. Boulard et al. focusing on the whole macroH2A1 gene (termed *H2AFY*), showed that ~ 50% of KO females specifically exhibited liver lipid homeostasis defects that led to hepatic steatosis^34^. MacroH2A1 was specifically enriched in nucleosomes that occupy the X-linked thyroxine-binding globulin (Tbg) promoter in female hepatocytes^34^. This study did not distinguish between the two exon-splicing isoforms (macroH2A1.1 and macroH2A1.2). In our macroH2A1.1 KO mice (where macroH2A1.2 is still expressed^30^) we did not detect differences in hepatic lipid accumulation, under a standard or a HFD diet, irrespective of the sex. It has been appreciated that differences in the genetic background (mixed vs. C57BL/6) could likely influence the penetrance of the hepatic phenotype in mice lacking the *H2AFY* gene ^34,49^. Gut microbiota are an important component of normal physiology and there is an intimate crosstalk between the microbial world in the gut and the host. Interestingly, both host histones released in the bloodstream and cell free bacterial DNA activate immune processes through Toll Like Receptor signaling^53^. Chromatin dynamics of the host epithelium involving histone modifications and histone variants and other parts of the epigenetic machinery play a crucial role in this process^54^. For instance, microbiota-derived metabolite butyrate inhibits the activity of histone deacetylase 3 (HDAC3) in the gut^55,56^. Generally, there are significant differences in the gut microbiota in male and female mice^57^. At baseline, female, but not male, macroH2A1.1 KO mice displayed an increased alpha diversity; HFD itself has been reported to increase alpha diversity in mice before the onset of obesity^58,59^. In particular, our female macroH2A1.1 KO mice exhibited an increase in Firmicutes (F) and a decrease in Bacteroidetes (B), which was not observed in male mice; this shift in the F/B ratio is consistently observed in obese subjects^46,47^. The dysbiotic gut microbiota of female macroH2A1.1 KO mice might help to explain their predisposition to develop obesity. The F/B ratio has also an important influence in maintaining normal intestinal homeostasis. Altered F/B ratios may predispose to inflammatory bowel disease (IBD)^60^. Interestingly, Cedeno et al. demonstrated that mice lacking all macroH2A proteins (including macroH2A1.1 and macroH2A1.2) exhibited an impaired reserve intestinal stem cell number and function during homeostasis and regeneration^61^, a feature of IBD. However, this study did not investigate sexual dimorphism. MacroH2A1 isoforms play a role in both transcriptional repression and activation^18,62–64^. Many studies indicated that macroH2A1 dynamic recruitment and enrichment onto the Xi upon inactivation in female mammals could reflect a possible function in sex-chromosome dosage compensation and genome stability^65–69^. However, quantities of macroH2A1 variant transcripts (macroH2A1.1 and macroH2A1.2) were similar in the tissues of male and female mice^70^. In gonadal male and female mice carrying XX or XY sex chromosome complements, a subset of genes that escape Xi exhibited higher expression levels in adipose tissue and liver and contributed to worsened obesity and dyslipidemia in females^71–73^. Interestingly, Changolkar et al. reported sexual dimorphic effects of macroH2A1 KO (without distinguishing between macroH2A1.1 and macroH2A1.2 isoforms) on the expression of 6 lipid metabolizing genes (Serpina7, Lpl, Krt1-23, ATP11a, Scd2, CD36) in mice^32^. Interestingly, they reported also that male macroH2A1 KO (for both macroH2A1.1 and macroH2A1.2), but not female mice, displayed a worsened glucose tolerance compared to their wild type littermates females at baseline^32^. While we observed a similar non statistically significant trend at the baseline, our macroH2A1.1 KO male mice in the same C57BL/6 genetic background appear more resistant than their wild type littermates to develop glucose intolerance upon a HFD, suggesting that the systemic presence of macroH2A1.2 isoform could be conferring metabolic protection more pronounced in males than in females. In terms of fat accumulation, pro-adipogenic and anti-adipogenic roles for macroH2A1.1 and macroH2A1.2, respectively, were consistently demonstrated in parallel in in vitro setting^27,51^. MacroH2A1.1 and macroH2A1.2 differential metabolic effects on glucose and lipid turnover remain to be systematically studied in vivo in the same genetic background in the two genders, taking into account eventual compensatory effects in isoforms expression^69^. Changolkar et al. later showed that macroH2A1 is enriched on the transcribed regions of 5 of these 6 genes by performing a chromosome-wide assessment of genes that escape from Xi^63^. In our macroH2A1.1 mouse model the link between altered expression of macroH2A1 isoforms, Xi and obesity deserves to be further explored, as it might also be underlying the sexual dimorphism in gut microbiota composition and in the female bias of autoimmune diseases^74^. MacroH2A1 variants impact importantly the transcriptional regulation of tumorigenesis of many cancer types, notably gastrointestinal (GI) ones, as we and others have extensively reported^62,75–77^. Obesity is a significant risk factor for developing cancer in the GI tract^78,79^, which in turn displays significant sexual dimorphism in their incidence in human populations^80^. In conclusion, macroH2A1.1 KO mice display sexual dimorphism in HFD-induced obesity and in gut dysbiosis and may represent a useful model to investigate epigenetic and metabolic differences associated to the development of obesity-associated pathological conditions in males and females.

### Supplementary Information

Supplementary Information 1.

Supplementary Information 2.

Supplementary Information 3.

## Data Availability

The metagenomic datasets generated and/or analysed during the current study are available in the ENA repository, under the accession number PRJEB62479, secondary accession ERP147570.
