# Supplementary material for: Deficiency of histone variant macroH2A1.1 is associated with sexually dimorphic obesity in mice
Source: Sci Rep. 2023 Nov 5;13:19123. doi: 10.1038/s41598-023-46304-8 (PMC10625986; doi:10.1038/s41598-023-46304-8)
Supplement: Supplementary file 2 — Supplementary Information 2. [file 41598_2023_46304_MOESM2_ESM.docx]

Supplementary Table 1: complete statistical summary analysis

| Analyses | Measurement | Statistical Test | Comparison | Statistics | Degrees of Freedom | P | Fig |
| --- | --- | --- | --- | --- | --- | --- | --- |
| Weight gain | % of the basal (male) | Two-Way ANOVA | Factor 1-Genotype | F=0.0054 | 1, 33 | p=0.9415 | 1B |
|  |  |  | Factor 2-Diet | F=51.12 | 1, 33 | p<0.0001 |  |
|  |  |  | Interaction  (F1 x F2) | F=1.269 | 1, 33 | p=0.2681 |  |
|  | % of the basal (female) | Two-Way ANOVA | Factor 1-Genotype | F=8.657 | 1, 29 | p=0.0063 | 1B |
|  |  |  | Factor 2-Diet | F=22.60 | 1, 29 | p<0.0001 |  |
|  |  |  | Interaction  (F1 x F2) | F=0.2645 | 1, 29 | P=0.6109 |  |
|  |  | PLSD  Post-hoc Test | F1/F1-HFD vs  F1/F1-SD |  |  | p<0.05 |  |
|  |  |  | KO-HFD vs  KO-SD |  |  | p<0.001 |  |
|  |  |  | KO-HFD vs  F1/F1-HFD |  |  | p<0.05 |  |
| Food Intake | g/day (male) | Two-Way ANOVA | Factor 1-Genotype | F=108.9 | 1, 218 | p<0.0001 | 1C |
|  |  |  | Factor 2-Diet | F=258.2 | 1, 218 | p<0.0001 |  |
|  |  |  | Interaction  (F1 x F2) | F=0.2362 | 1, 218 | p=0.6275 |  |
|  | g/day (female) | Two-Way ANOVA | Factor 1-Genotype | F=6.144 | 1, 143 | p=0.00143 | 1C |
|  |  |  | Factor 2-Diet | F=198.9 | 1, 143 | p<0.0001 |  |
|  |  |  | Interaction  (F1 x F2) | F=4.357 | 1, 143 | p=0.0386 |  |
|  |  | PLSD  Post-hoc Test | KO-HFD vs  KO-SD |  |  | p<0.0001 |  |
|  |  |  | F1/F1-HFD vs  F1/F1-SD |  |  | p<0.0001 |  |
|  |  |  | KO-SD vs  F1/F1-SD |  |  | p<0.05 |  |
|  |  |  | KO-HFD vs  F1/F1-HFD |  |  | p>0.05 |  |
