# Supplementary material for: Deficiency of histone variant macroH2A1.1 is associated with sexually dimorphic obesity in mice
Source: Sci Rep. 2023 Nov 5;13:19123. doi: 10.1038/s41598-023-46304-8 (PMC10625986; doi:10.1038/s41598-023-46304-8)
Supplement: Supplementary file 3 — Supplementary Information 3. [file 41598_2023_46304_MOESM3_ESM.docx]

## Supplementary Table 2. Adipogenic gene expression in male/female macroH2A1.1 KO mice fed a HFD.

Values represent means of fold regulation of 3 mice per condition (female macroH2A1.1 KO and male macroH2A1.1 KO mice) compared to their respective control (Fl/Fl) mice.

In red = genes upregulated > 2 fold compared to Fl/Fl mice of the respective gender.

In blue = genes downregulated > 2 fold compared to Fl/Fl mice of the respective gender.

P-values adjusted for multiple testing using false discovery rate method (in bold <0.01).

| **RefSeq** | | **Symbol** | **Female macroH2A1.1 KO + HFD**  **Fold changes** | **Adjusted p value** | **Male macroH2A1.1 KO + HFD**  **Fold changes** | | | **Adjusted p value** | |
| --- | --- | --- | --- | --- | --- | --- | --- | --- | --- |
| NM_133904 | | Acacb | 3.054 | **0.00012** | 0.942 | | | 0.08712 | |
| NM_145635 | | Adig | 1.378 | 0.29742 | 1.103 | | | 0.67025 | |
| NM_009605 | | Adipoq | 1.034 | 0.52033 | 0.596 | | | 0.33815 | |
| NM_007420 | | Adrb2 | 0.490 | 0.31156 | 0.947 | | | 0.21119 | |
| NM_007428 | | Agt | 3.405 | **0.00022** | 1.675 | | | 0.35953 | |
| NM_007426 | | Angpt2 | 14.506 | **1.50E-12** | 2.007 | | | **0.00434** | |
| NM_009733 | | Axin1 | -10.932 | **6.70E-09** | -2.056 | | | **0.00703** | |
| NM_007553 | | Bmp2 | 1.345 | 0.05981 | 1.424 | | | 0.30264 | |
| NM_007554 | | Bmp4 | 0.670 | 0.09406 | 1.120 | | | 0.29672 | |
| NM_007557 | | Bmp7 | 1.128 | 0.10965 | 1.006 | | | 0.39670 | |
| NM_007631 | | Ccnd1 | -3.207 | **0.00341** | 1.678 | | | 0.43392 | |
| NM_009870 | | Cdk4 | -2.807 | **0.00416** | 1.473 | | | 0.26307 | |
| NM_007669 | | Cdkn1a | -2.792 | **0.00945** | -2.452 | | | **0.00762** | |
| NM_009875 | | Cdkn1b | -2.518 | **0.00017** | 1.429 | | | 0.55048 | |
| NM_007678 | | Cebpa | 0.792 | 0.45073 | 0.930 | | | 0.69382 | |
| NM_009883 | | Cebpb | 1.606 | 0.60241 | 1.128 | | | 0.74029 | |
| NM_007679 | | Cebpd | 0.403 | 0.08305 | 0.508 | | | 0.83201 | |
| NM_013459 | | Cfd | 1.495 | 0.30254 | 1.303 | | | 0.70462 | |
| NM_133828 | | Creb1 | 1.322 | 0.08594 | 1.209 | | | 0.49036 | |
| NM_007837 | | Ddit3 | 0.707 | 0.30266 | 0.603 | | | 0.70330 | |
| NM_010050 | | Dio2 | 1.508 | 0.10295 | 1.188 | | | 0.19011 | |
| NM_010051 | | Dkk1 | 1.783 | 0.30596 | 1.842 | | | **0.00893** | |
| NM_010052 | | Dlk1 | 0.306 | 0.78942 | 0.403 | | | 0.35820 | |
| NM_007891 | | E2f1 | -4.723 | 0.00002 | 1.123 | | | 0.06932 | |
| NM_010118 | | Egr2 | -11.408 | **2.45E-08** | -2.778 | | | **0.00946** | |
| NM_024406 | | Fabp4 | -1.6703 | **0.00406** | -0.938 | | | 0.10598 | |
| NM_007988 | | Fasn | 8.790 | **1.27E-07** | 3.530 | | | **0.00033** | |
| NM_010197 | | Fgf1 | 0.459 | 0.30295 | 0.396 | | | 0.29587 | |
| NM_008002 | | Fgf10 | 0.102 | 0.80035 | 0.284 | | | 0.42102 | |
| NM_008006 | | Fgf2 | -0.498 | 0.59604 | -0.320 | | | 0.40956 | |
| NM_013519 | | Foxc2 | 1.503 | 0.20951 | 1.305 | | | 0.03491 | |
| NM_019739 | | Foxo1 | -0.893 | 0.08322 | -0.566 | | | 0.12873 | |
| NM_008090 | | Gata2 | 0.424 | 0.39281 | 0.387 | | | 0.05042 | |
| NM_008091 | | Gata3 | -0.288 | 0.67920 | -0.166 | | | 0.39510 | |
| NM_008235 | | Hes1 | 1.401 | 0.40698 | 1.205 | | | 0.59063 | |
| NM_010568 | | Insr | -1.095 | 0.30507 | -0.941 | | | 0.55001 | |
| NM_010570 | | Irs1 | -1.105 | 0.40195 | -1.006 | | | 0.03958 | |
| NM_001081212 | | Irs2 | -0.780 | 0.55002 | -0.430 | | | 0.39303 | |
| NM_010591 | | Jun | -2.503 | **0.00193** | 1.502 | | | 0.67022 | |
| NM_023184 | | Klf15 | -1.306 | 0.56942 | 0.535 | | | 0.49306 | |
| NM_008452 | | Klf2 | -0.833 | 0.60374 | -0.679 | | | 0.53055 | |
| NM_008453 | | Klf3 | -1.562 | 0.04862 | -0.938 | | | 0.43024 | |
| NM_010637 | | Klf4 | -0.590 | 0.30295 | -0.388 | | | 0.26670 | |
| NM_008493 | | Lep | 1.322 | 0.10884 | 1.034 | | | 0.33775 | |
| NM_010719 | | Lipe | 0.711 | 0.85012 | 0.593 | | | 0.77392 | |
| NM_019390 | Lmna | | 1.257 | 0.47782 | 1.034 | | 0.80351 | |  |
| NM_008509 | Lpl | | 0.478 | 0.66011 | 0.607 | 0.33082 | | |  |
| NM_008513 | Lrp5 | | 1.157 | 0.37002 | 1.006 | 0.44033 | | |  |
| NM_011951 | Mapk14 | | -1.709 | 0.15084 | -1.328 | 0.09360 | | |  |
| NM_008678 | Ncoa2 | | 0.588 | 0.30022 | 0.452 | 0.44096 | | |  |
| NM_011424 | Ncor2 | | -1.204 | 0.46600 | -1.122 | 0.64730 | | |  |
| NM_011850 | Nr0b2 | | 0.565 | 0.49268 | 0.320 | 0.75004 | | |  |
| NM_013839 | Nr1h3 | | 1.123 | 0.62022 | 1.326 | 0.83053 | | |  |
| NM_010938 | Nrf1 | | 0.674 | 0.53073 | 1.435 | 0.33291 | | |  |
| NM_011144 | Ppara | | 1.034 | 0.22508 | 0.607 | 0.44020 | | |  |
| NM_011145 | Ppard | | -0.778 | 0.33025 | -0.824 | 0.56293 | | |  |
| NM_011146 | Pparg | | 1.479 | 0.22905 | 1.208 | 0.33206 | | |  |
| NM_008904 | Ppargc1a | | 0.563 | 0.20784 | 0.306 | 0.50043 | | |  |
| NM_133249 | Ppargc1b | | 1.306 | 0.33895 | 1.280 | 0.40366 | | |  |
| NM_027504 | Prdm16 | | 1.402 | 0.53092 | 1.649 | 0.55660 | | |  |
| NM_009029 | Rb1 | | 0.329 | 0.67784 | 0.310 | 0.30995 | | |  |
| NM_022984 | Retn | | 4.048 | 8.50E-09 | 2.654 | 0.00028 | | |  |
| NM_009822 | Runx1t1 | | -1.305 | 0.30030 | 0.884 | 0.29583 | | |  |
| NM_011305 | Rxra | | 1.545 | 0.32006 | 1.005 | 0.20953 | | |  |
| NM_013834 | Sfrp1 | | -0.583 | 0.55039 | -0.435 | 0.69482 | | |  |
| NM_018780 | Sfrp5 | | -1.108 | 0.42952 | -0.793 | 0.88341 | | |  |
| NM_009170 | Shh | | 0.785 | 0.38390 | 0.639 | 0.22115 | | |  |
| NM_019812 | Sirt1 | | -2.304 | 0.00250 | 1.502 | 0.35415 | | |  |
| NM_022432 | Sirt2 | | -2.004 | 0.00679 | 1.399 | 0.52883 | | |  |
| NM_022433 | Sirt3 | | -1.749 | 0.06381 | 1.057 | 0.44983 | | |  |
| NM_009204 | Slc2a4 | | 4.532 | 0.00034 | 2.302 | 0.00512 | | |  |
| NM_009271 | Src | | 1.758 | 0.28854 | 1.205 | 0.19992 | | |  |
| NM_011480 | Srebf1 | | -1.597 | 0.09458 | -1.345 | 0.10063 | | |  |
| NM_181516 | Taz | | 0.506 | 0.59973 | 0.404 | 0.12237 | | |  |
| NM_009333 | Tcf7l2 | | 1.005 | 0.22995 | 0.884 | 0.33506 | | |  |
| NM_010286 | Tsc22d3 | | -0.563 | 0.39436 | -0.128 | 0.66053 | | |  |
| NM_011658 | Twist1 | | 1.005 | 0.59053 | -0.252 | 0.88025 | | |  |
| NM_009463 | Ucp1 | | -5.107 | 7.80E-07 | 1.354 | 0.43910 | | |  |
| NM_009504 | Vdr | | 1.007 | 0.55904 | 1.195 | 0.30345 | | |  |
| NM_021279 | Wnt1 | | -1.560 | 0.09483 | -1.504 | 0.11022 | | |  |
| NM_011718 | Wnt10b | | -2.004 | 0.00735 | -0.589 | 0.33945 | | |  |
| NM_009522 | Wnt3a | | -1.783 | 0.11884 | -0.951 | 0.83488 | | |  |
| NM_009524 | Wnt5a | | -1.236 | 0.08573 | 0.884 | 0.71124 | | |  |
| NM_009525 | Wnt5b | | -1.596 | 0.10038 | -1.066 | 0.387500 | | |  |
